# Current Status and Trends in mHealth-Based Research for Treatment and Intervention in Tinnitus: Bibliometric and Comparative Product Analysis

**DOI:** 10.2196/47553

**Published:** 2023-08-24

**Authors:** Yuanjia Hu, Yang Lu, Chenghua Tian, Yunfan He, Kaiyi Rong, Sijia Pan, Jianbo Lei

**Affiliations:** 1 School of Medical Technology and Information Engineering, Zhejiang Chinese Medical University Hang Zhou China; 2 School of Public Health, Zhejiang University Hangzhou China; 3 The Second School of Clinical Medicine, Zhejiang Chinese Medical University Hangzhou China; 4 Center for Medical Informatics, Peking University Beijing China; 5 Institute of Medical Technology, Peking University Beijing China; 6 School of Medical Informatics and Engineering, Southwest Medical University Luzhou China

**Keywords:** tinnitus, mobile health, mHealth, internet, application, software, bibliometrics, mobile phone

## Abstract

**Background:**

As a global medical problem, tinnitus can seriously harm human health and is difficult to alleviate, ranking among the top 3 complex diseases in the otolaryngology field. Traditional cognitive behavioral therapy and sound therapy require offline face-to-face treatment with medical staff and have limited effectiveness. Mobile health (mHealth), which, in recent decades, has been greatly applied in the field of rehabilitation health care, improving access to health care resources and the quality of services, has potential research value in the adjunctive treatment of tinnitus.

**Objective:**

This study aimed to understand the research trends, product characteristics, problems, and research transformation of tinnitus treatment software by analyzing the research progress of mHealth for tinnitus treatment based on the literature and related marketed apps.

**Methods:**

Bibliometric methods were used to describe the characteristics of the relevant literature in terms of the number and topics of publications, authors, and institutions. We further compared the features and limitations of the currently available tinnitus treatment software.

**Results:**

Data published until February 28, 2022, were collected. Following the PRISMA (Preferred Reporting Items for Systematic Reviews and Meta-Analyses) standardized screening process, 75 papers were included. The country with the highest number of publications was Germany, followed by the United Kingdom and the United States, whereas China had only a single relevant study. The most frequently found journals were the *American Journal of Audiology* and the *Journal of the American Academy of Audiology* (18/75, 24%). With regard to publication topics, cognitive behavioral therapy started to become a hot topic in 2017, and research on mHealth apps has increased. In this study, 28 tinnitus treatment apps were obtained (n=24, 86% from product data and n=4, 14% from literature data); these apps were developed mainly in the United States (10/28, 36%) or China (9/28, 32%). The main treatment methods were sound therapy (10/28, 36%) and cognitive behavioral therapy (2/28, 7%). Of the 75 publications, 7 (9%) described apps in the market stage. Of the 28 apps, 22 (79%) lacked literature studies or evidence from professional bodies.

**Conclusions:**

We found that, as a whole, the use of mHealth for treatment and intervention in tinnitus was showing a rapid development, in which good progress had been made in studies around sound therapy and cognitive behavioral therapy, although most of the studies (50/75, 67%) focused on treatment effects. However, the field is poorly accepted in top medical journals, and the majority are in the research design phase, with a lack of translation of the literature results and clinical validation of the marketed apps. Furthermore, in the future, novel artificial intelligence techniques should be used to address the issue of staged monitoring of tinnitus.

## Introduction

### Background

The American Academy of Otolaryngology-Head and Neck Surgery defines tinnitus as “a sound perceived by the patient in the absence of an external sound source” [1]. Several studies have shown that the worldwide prevalence rate of tinnitus ranges from 5.1% to 42.7% [2]. Xu et al [3] showed that 9.6% of the global adult population has experienced tinnitus in the past 12 months. Of these, 36% had persistent tinnitus, and 27% had tinnitus for >15 years. Persistent tinnitus can lead to severe psychological disorders, such as depression, anxiety, mania, and other affective disorders. Suicide and personal injury rates are significantly increased in patients with tinnitus [4]. As a matter of concern, the decreased availability of effective treatments for tinnitus greatly increases the socioeconomic burden of this disease in medical care [5].

Tinnitus lacks effective standardized and individualized treatments. Drugs, surgery, electrical stimulation, and psychophysiological integrative therapy are traditional treatment modalities that have serious adverse effects. Moreover, their long-term safety is unknown. Mobile health (mHealth) apps are a novel tool expected to be effective in alleviating tinnitus. The popularity of smartphones and the inherent immediacy, accuracy, and low costs of the mobile internet have contributed to the unique advantages of mHealth apps in providing health interventions and other outcomes. As of 2020, there were >300,000 (this number is rapidly growing) mHealth apps available in mobile app stores [6]. These apps help users to monitor multiple physiological indicators and provide relevant health knowledge and services to address a range of health issues [7], including tinnitus. Several studies have found that mHealth apps can provide continuous and remote monitoring of tinnitus as well as diagnostic and intervention services for patients with the condition. This can, in turn, be effective in alleviating or resolving this disorder. Most Chinese researchers provide sound therapy for patients with tinnitus through software. There are also attempts to use herbal medicine, acupuncture, and electrical stimulation for the same purpose. He et al [8] used audition software for sound therapy. Cai et al [9] integrated personalized music into tinnitus software, in combination with cognitive behavioral therapy, to innovate a tinnitus treatment intervention with substantial results.

### Objectives

Nevertheless, most studies have focused only on monotherapeutic interventions for tinnitus. As such, there is a lack of a systematic analysis of tinnitus treatment software studies and marketed apps [10]. Therefore, using bibliometric and comparative product analysis methods, this study provides a complete and detailed description of the field of tinnitus monitoring, diagnosis, and intervention with the help of mHealth apps. Our work is the first bibliometric study to comprehensively analyze trends in publication, national and institutional distribution, core journals and highly productive authors, and research topic hot spots in this area. This study aimed to understand the research trends in tinnitus treatment software, including the characteristics and limitations of the apps available in the market. The outputs of our research can help patients to choose the right tinnitus treatment software and provide suggestions to manufacturers regarding potential ways to improve the quality and use of tinnitus treatment software.

## Methods

### Data Retrieval and Filtering

Regarding the research literature, to ensure completeness, our study followed the PRISMA (Preferred Reporting Items for Systematic Reviews and Meta-Analyses) principle and searched databases in 5 different fields [11,12]. In the medical field, the PubMed and Embase databases were used. In the general science field, the Web of Science core collection was chosen. In the computer field, the IEEE and ACM databases were chosen. The search queries and strategies used for the different databases are listed in Multimedia Appendix 1. The inclusion and exclusion criteria for this study are shown in Multimedia Appendix 2.

**Figure 1:**
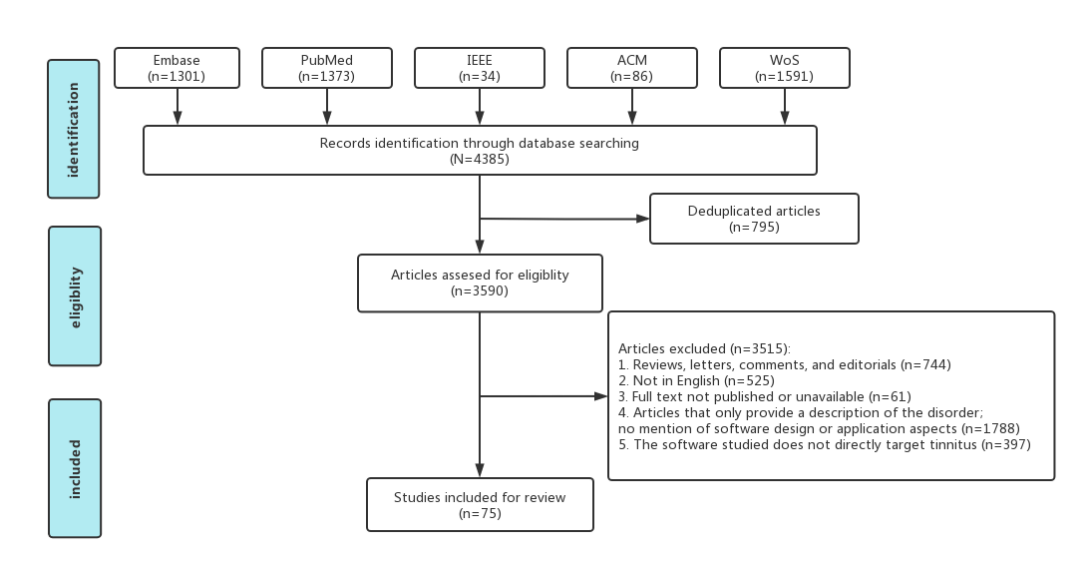
Flowchart of literature data screening. WoS: Web of Science.

A total of 4385 papers were retrieved from 5 databases (n=1301, 29.67% from Embase; n=34, 0.78% from IEEE; n=1373, 31.31% from PubMed; n=1591, 36.28% from Web of Science; and n=86, 1.96% from ACM). After literature deduplication, of the 4385 papers, 3590 (81.87%) remained. Two trained postgraduate students screened the retrieved papers according to the established inclusion and exclusion criteria (Cohen κ=0.91), and, of the 3590 papers, 75 (2.09%) were included in the final literature pool, as illustrated in Figure 1.

After a preliminary market survey, we identified 2 sources of information in which to search for product data: the Android platform and the Apple App Store (China and the United States), for a total of 7 official mobile software marketplaces, with the Android platform accounting for 5 (71%) mobile software marketplaces (Google Play Store as well as the mobile software marketplaces operated by Huawei, Vivo, Oppo, and Xiaomi). To obtain relevant search results for mHealth apps on the Qimai app data analysis platform [13] (accessed in March 2021), we used the following search terms: “tinnitus diagnosis,” “tinnitus intervention,” and “tinnitus treatment” A total of 2384 mHealth apps were retrieved, with 1812 (76%) stand-alone mHealth apps remaining after data deduplication.

**Figure 2:**
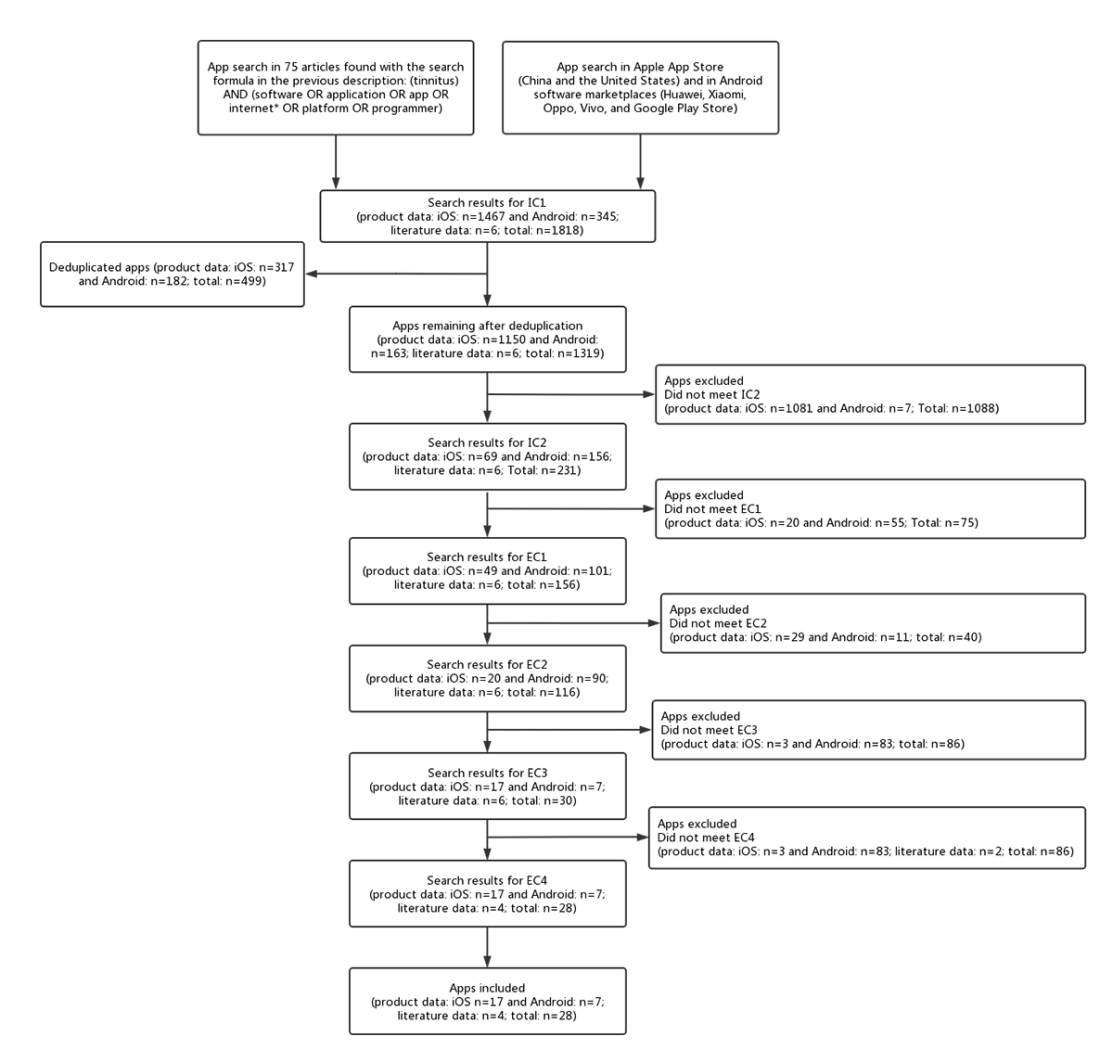
The screening flowchart of the mobile health apps. EC1: exclusion criterion 1; EC2: exclusion criterion 2; EC3: exclusion criterion 3; EC4: exclusion criterion 4; IC1: inclusion criterion 1; IC2: inclusion criterion 2.

In this study, mHealth apps related to tinnitus treatment were screened from 75 literature reports and 1812 independent software reports, using established inclusion and exclusion criteria. A total of 28 tinnitus treatment apps were obtained (n=24, 86% from product data and n=4, 14% from literature data). The specific inclusion and exclusion criteria applied to select the target apps are listed in Textbox 1. A screening flowchart of the mHealth apps is shown in Figure 2.

**Inclusion and exclusion criteria applied to select the target apps.**

**Inclusion criteria**

- Inclusion criterion (IC) 1: apps identified with the search terms “tinnitus,” “hearing test,” “sound therapy,” “cognitive behavioural therapy,” “cbt,” “perceptual mask,” “perceptual masking,” “retraining therapy,” and “sleep”
- IC2: apps with either a health purpose or a medical purpose

**Exclusion criteria**

- Exclusion criterion (EC) 1: Apps consisting only of sleep aid, relaxation, or meditation software; pure mood journals; or diary functionalities (with or without cognitive behavioral therapy)
- EC2: apps consisting only of mobile hearing test, aid, or debugging software, pure mobile sound level meter software, or pure speech therapy software (not related to tinnitus)
- EC3: health management software for other diseases (not related to tinnitus)
- EC4: insufficient software information

### Extraction of App Information

In this study, we extracted general and specific information about 28 apps, using the Qimai app data analysis platform. General information about the apps included their names, reviews, and ratings. The specific information obtained on the apps was analyzed from 3 perspectives: time, space, and content. From a temporal perspective, the fields extracted from the specific information included the dates of product release and last update, as well as in-app, off-app, and unreleased stores. With regard to the spatial perspective, the specific information included the product’s country of origin and the name, location, and size of the product manufacturer. From the content perspective, the specific information included type, purpose, input and output information, clinical role, main treatments related to the software, their effectiveness, and software scenario.

### Data Analysis Methods

**Figure 3:**
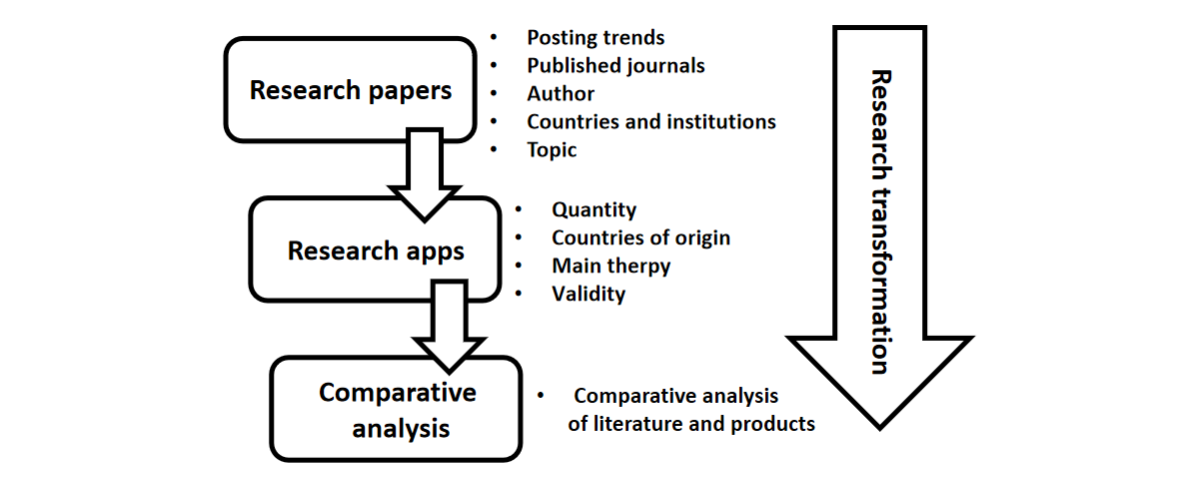
Analysis chain and process.

To review and summarize the current status of, and research trends in, mHealth treatments and interventions for tinnitus, we used a bibliometric and comparative product analysis approach, based on a *literature output-product output–literature versus product* chain. We used the *bibliometrix* package in R for data analysis and the *ggplot2* package for mapping. First, a bibliometric approach was used to analyze the literature data and obtain the current publication outputs. Second, product data were analyzed to obtain information about product outputs and applications. Finally, we compared the development of literature and product components. The purpose was to obtain the differences between the current state of research and its actual use, as well as to gain a preliminary insight into the translation of research in this field. The analysis chain and process are shown in Figure 3.

## Results

### Analysis of the Literature Output

Regarding the literature output, we analyzed the retrieved articles using bibliometric methods concerning 5 aspects: publication trends, journals, authors, countries and institutions, and themes.

#### Analysis of Publication Trends

As of February 2022, a total of 162 authors had published 75 relevant articles in 44 different journals. Among the 162 authors, 46 (28.4%) were lead authors. In addition, there were an average of 0.11 (SD 0.98) relevant articles per journal per year and an average of 0.46 (SD 1.27) articles per author. The evolution in the annual number of relevant studies published over the years is shown in Multimedia Appendix 3. With regard to the 75 articles included in this study, there was an overall increasing trend in the annual number of literature publications. There was an average annual increase rate of 24.46% (SD 0.69%), which resulted in 16 articles published in 2021. This trend demonstrates that an increasing amount of research is being carried out on this topic, indicating the increasing need for research in this field.

There was a substantially increased number of articles published in 2015 and 2021, in comparison with the previous and subsequent years. The authors of the 9 articles published in 2015 were mainly from Sweden and the United Kingdom, accounting for 3 (33%) and 2 (22%) articles, respectively. There were 4 articles by Gerhard Andersson as the lead author, from Linköping University (Linköping, Sweden), whose primary research interests were psychopathology and psychotherapy [14-17]. Of the 16 articles published in 2021, a total of 9 (56%) were from the United Kingdom, including 4 (44%) articles by Eldre Beukes, from Anglia Ruskin University, whose main research interest was audiology.

#### Analysis of the Major Publishing Journals

According to the Bradford law of scattering [18], all publications in a specific field, released during a given period, can be divided into core, related, or peripheral zones, according to the number of articles the zones include. The ratio of the number of publications in the 3 zones is 1:*a*:*a*^2^, with the approximate value of *a* being 5. Core publications account for approximately 1 of 31 of the total number of publications. The core publications were calculated to correspond to the top 2 journals, which accounted for 36% (18/75) of the published articles: the *American Journal of Audiology* (12/75, 16%) and the *Journal of the American Academy of Audiology* (6/75, 8%). In 2021, the journal impact factors of these 2 journals were 1.636 and 1.249, respectively, and the journal citation reports divisions were Q4 and Q3, respectively. The remaining publications revealed minor differences in terms of the number of published articles.

#### Analysis of Article Authors

There were 162 authors in the included literature. According to the Lotka law [19], which states that authors with >0.749 times the square root of the number of papers published by the most prolific scientists, this study defined authors with >4 publications as high-producing authors, accounting for 9.9% (16/162). As can be seen in Table 1, Andersson G was the most prolific author, with 22 publications.

**Figure 4:**
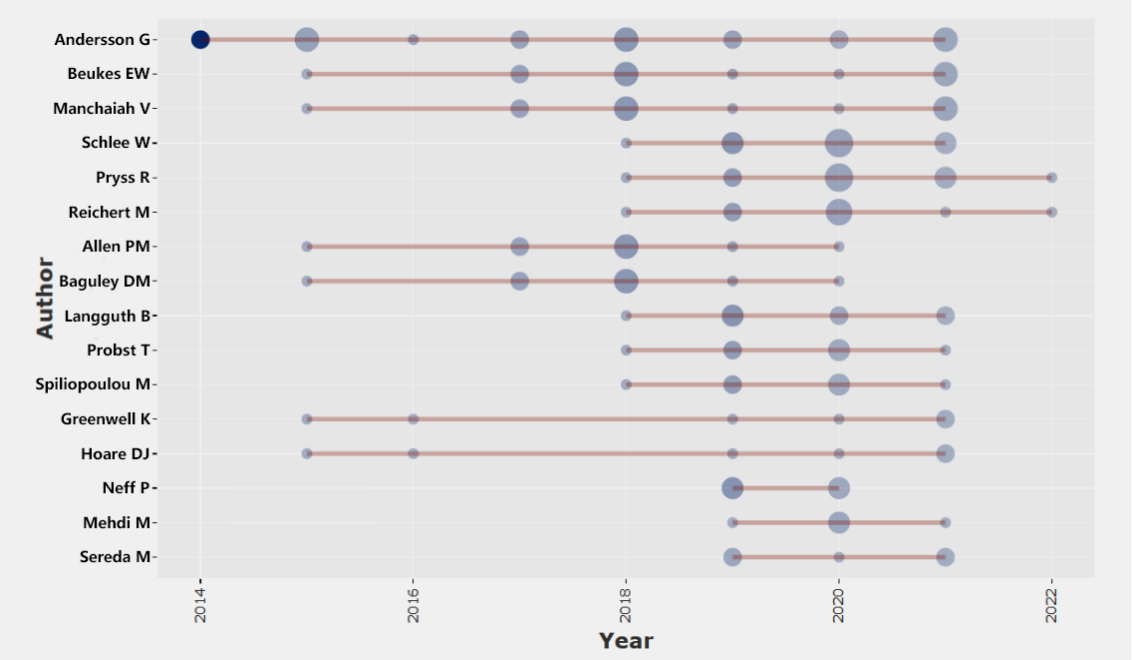
Productivity of the top authors over time.

Next, we performed an analysis of the posting trends from highly productive authors. A graph of the annual posting trends of highly productive authors, based on their annual posting data and yearly citation frequencies, is shown in Figure 4*.* Here, the size of the circles indicates the annual posting volume, and the color shade indicates the annual citation frequency. Of the 16 most productive authors, 15 (94%) were found to have started focusing on mHealth-based treatments and interventions for tinnitus only after 2015. Of these 15 authors, 9 (60%) started publishing after 2018, and they have produced a steady annual output since then.

**Table 1:** Ranking of authors with >4 publications.

Rank by number of articles	Author’s name	Articles (n=153), n (%)
1	Andersson G	22 (14.4)
2	Beukes EW	14 (9.2)
2	Manchaiah V	14 (9.2)
3	Schlee W	13 (8.5)
4	Pryss R	12 (7.8)
5	Reichert M	10 (6.5)
6	Allen PM	9 (5.9)
6	Baguley DM	9 (5.9)
7	Langguth B	8 (5.2)
8	Probst T	7 (4.6)
8	Spiliopoulou M	7 (4.6)
9	Greenwell K	6 (3.9)
9	Hoare DJ	6 (3.9)
9	Neff P	6 (3.9)
10	Mehdi M	5 (3.3)
10	Sereda M	5 (3.3)

#### Analysis of the Publication Country of Origin

**Figure 5:**
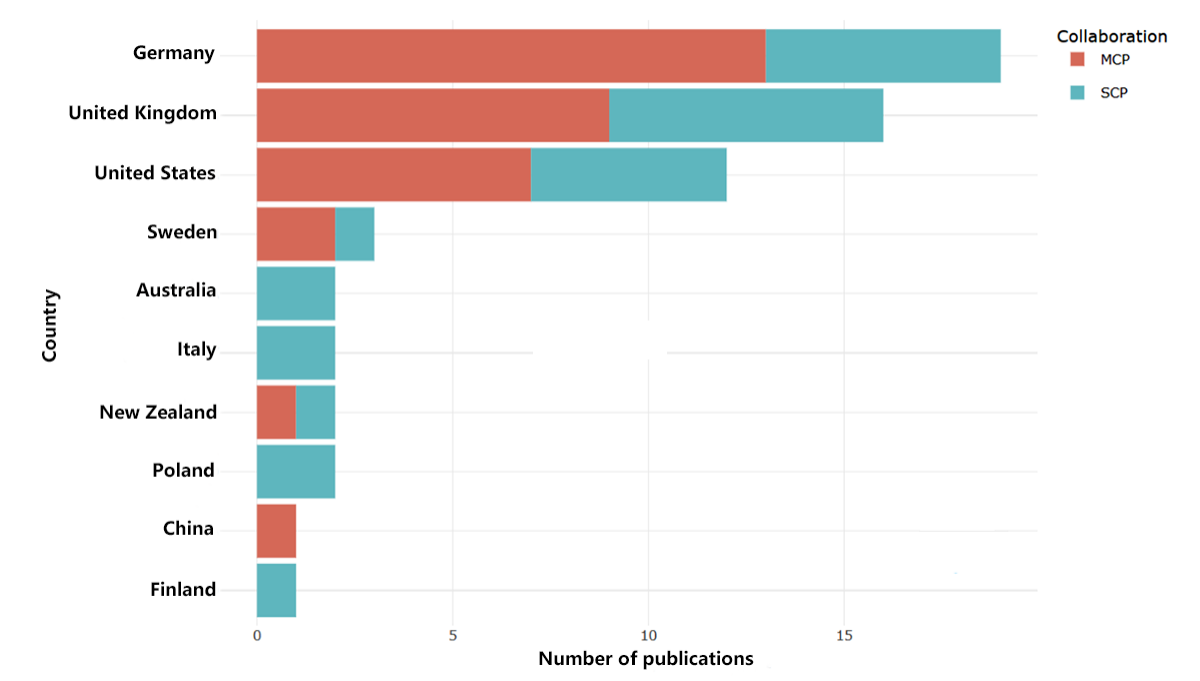
Articles by country of corresponding author. MCP: multicountry publication; SCP: single country publication.

The 162 authors in our literature pool were from 15 countries. The top 10 countries (corresponding authors’ countries) are shown in Figure 5. Germany has published the largest number of articles in this field (19/75, 25%) and also has the largest number of international collaborations, followed by the United Kingdom (16/75, 21%) and the United States (12/75, 16%). The numbers of multicountry publications and single country publications for the top 10 high-output countries are shown in Figure 5*.*

#### Analysis of Posting Themes

##### VOSviewer Keyword Clustering Analysis

In this study, we analyzed the themes of the publications in the field from 3 aspects. First, highly frequent keywords were extracted from the abstracts. These keywords referred to literature data, common aspects of highly frequent keywords, and their co-occurrence network. The main research themes were identified from the clustering results. Second, to explore current research hot spots, the thematic map method, proposed by Cobo et al [20], was used to cluster and map themes according to their density and centrality. Finally, trends in the evolution of themes in the field were analyzed from a time-course perspective.

**Figure 6:**
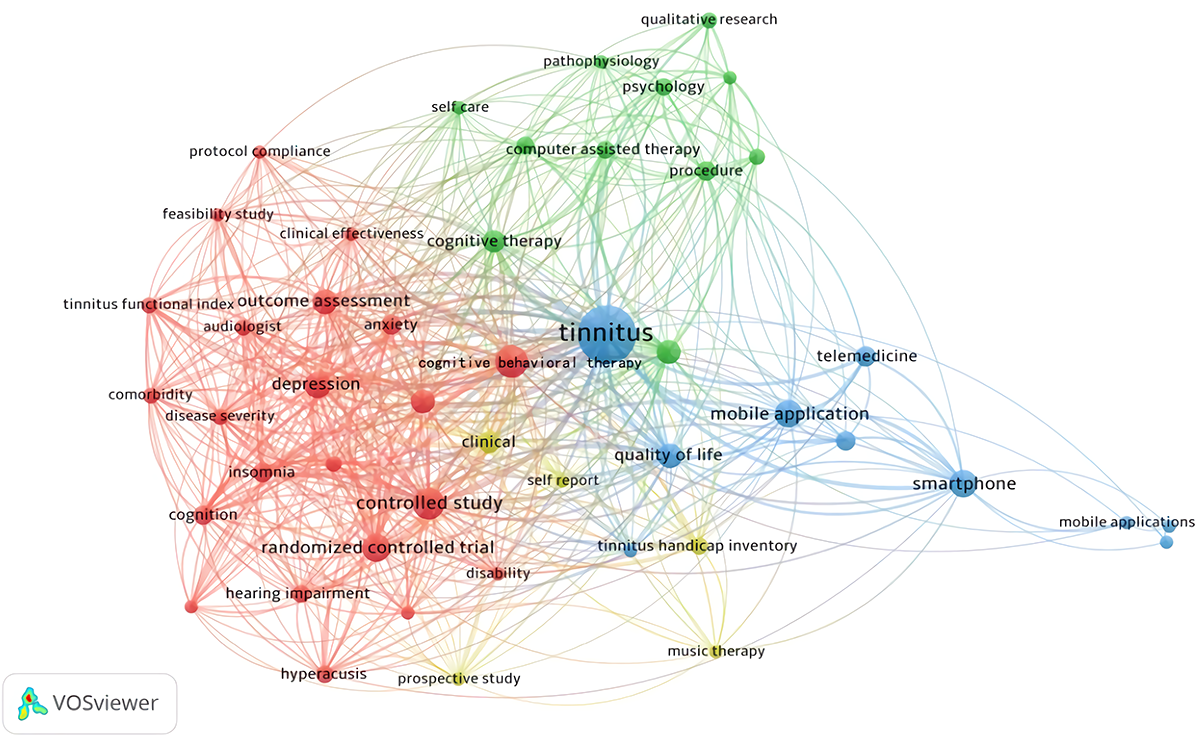
Keyword co-occurrence network diagram produced using VOSviewer [21].

Next, we conducted a visual analysis of highly frequent keyword co-occurrence networks. To obtain all keywords from the relevant studies, the abstracts of the included literature articles were divided, deactivated, and lexically normalized using Python (Python Software Foundation). As shown in Figure 6, a high-frequency keyword co-occurrence network was produced using VOSviewer [21]. As shown in this figure, clustering has divided all keywords into 4 main domains, which could be further combined into 3 categories: red and yellow, green, and blue. The red and yellow categories represent the main treatments currently available for tinnitus (including *cognitive behavioral therapy* and *music therapy*), as well as clinical trials and the main feelings experienced by patients with tinnitus. Clinical trial methods include *randomized controlled trials* and *clinical trials*. The subjective feelings of patients with tinnitus include *anxiety*, *depression*, and *insomnia*. The green category contains computer-related keywords such as *computer-assisted therapy and procedure*. By contrast, the blue category includes keywords such as *mobile app*, *smartphone*, and *telemedicine* for mHealth and mobile devices.

It was particularly interesting to note that *cognitive behavioral therapy* was used as a link between the words *disorder* and *computer*. The words linking *mHealth* and *computer* were mainly basic concepts such as *procedure*. The words linking *mHealth* and *disease* were mainly research methods such as *clinical study*, which were slightly less connected than the other 2 groups.

##### Thematic Map Analysis

Cobo et al [20] proposed the thematic map method, which uses the division of themes into quadrants to analyze the *hotness* and importance of a topic. Topics located in the first quadrant (niche themes) are well developed but relatively less important. Those in the second quadrant (motor themes) are well developed and essential. Topics located in the third quadrant (emerging or declining themes) are not well developed and relatively less compelling. The topics found in the fourth quadrant (basic themes) are not well developed but necessary and generally refer to basic concepts.

**Figure 7:**
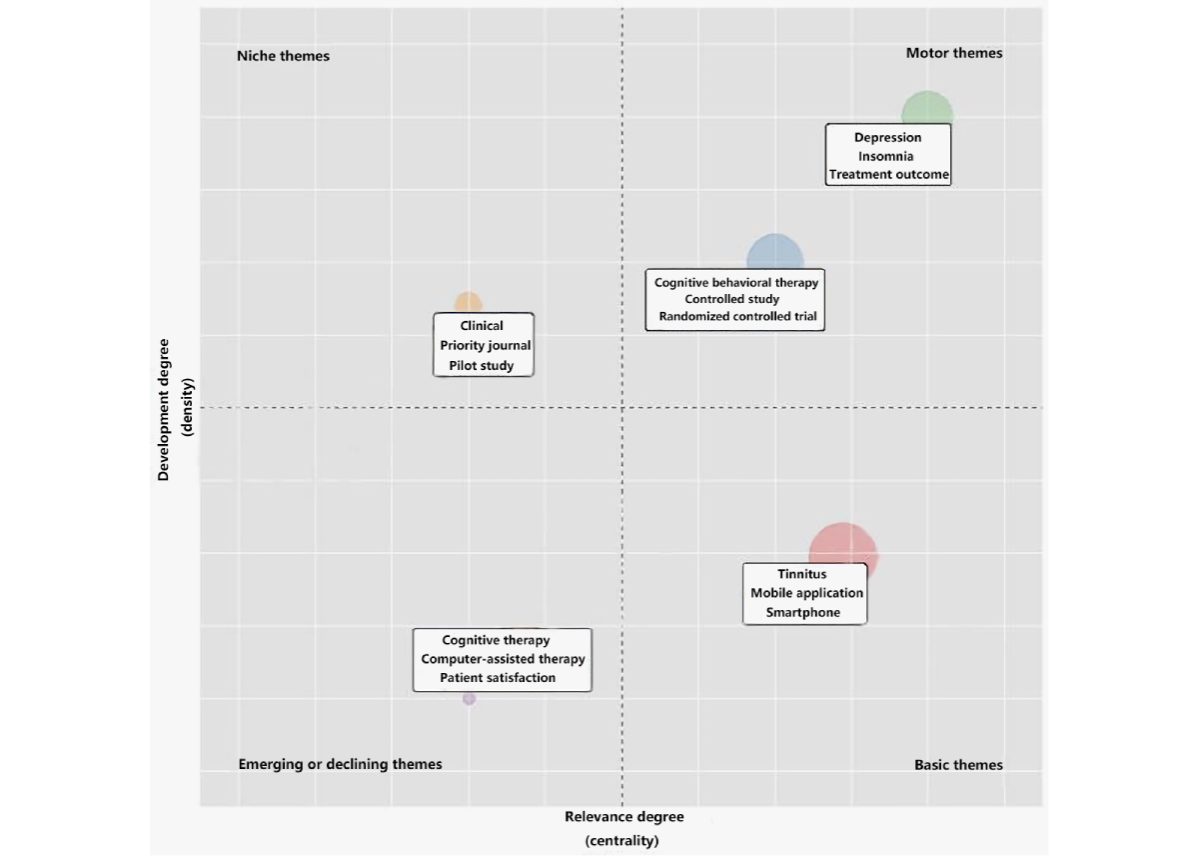
Thematic map with keywords divided into 4 domains.

By calculating the density and centrality of the already clustered coword matrix, each of the 5 categories was visualized within the 2D coordinates, as shown in Figure 7. We found that, in the first quadrant, the vocabulary of research methods (terms such as *clinical* and *pilot study*) was predominantly used, as well as concepts that have developed well and are currently being phased out. In the second quadrant, we observed that cognitive behavioral therapy and treatment outcomes were essential and well-studied topics. In the third quadrant, we observed that computer-assisted therapy was a topic that has not been well developed yet. In the fourth quadrant, as basic technologies in the field, the concepts of tinnitus, smartphones, and mobile apps were predominantly used.

##### Sankey Diagram Analysis

**Figure 8:**
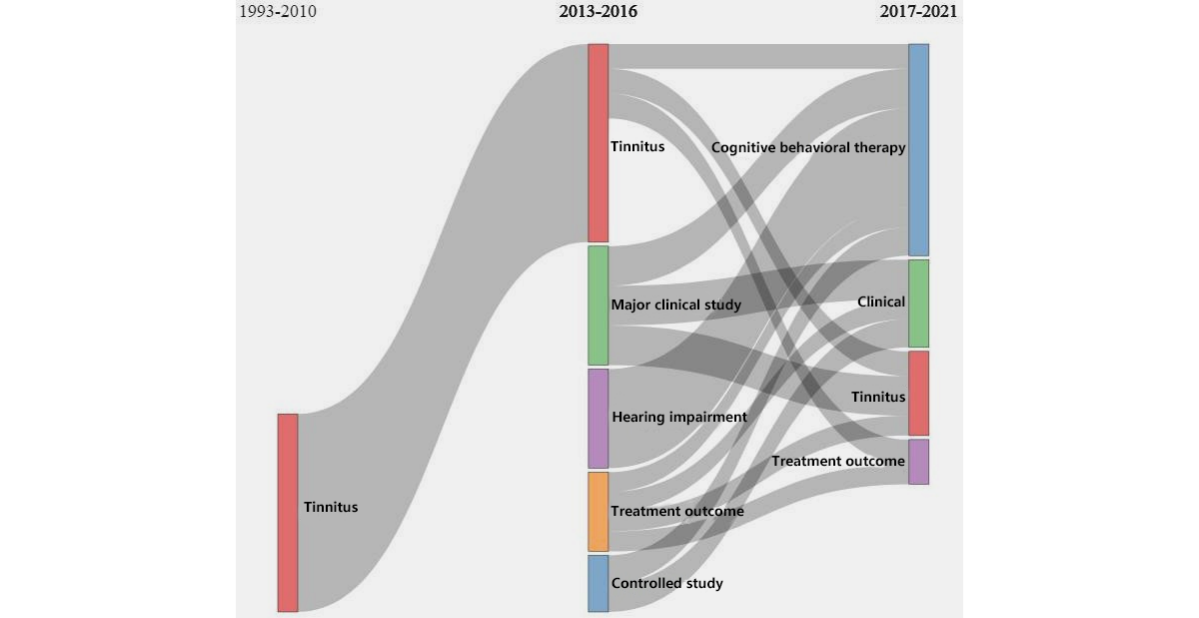
Sankey diagram of trends in the evolution of research themes.

A thematic evolution analysis was carried out using coword network analysis and clustering. This analysis incorporated the time dimension to analyze the evolution of the research themes from 1993 to 2021 and to create a Sankey diagram (Figure 8).

From this observation, it was clear that there were both changing and unchanging themes. Researchers from as early as the 1993-2010 period focused on tinnitus as a disorder, without introducing further meaningful concepts. During the 2013-2016 period, the traditional research themes did not entirely dissipate, but several research methods were added to these clinical studies. During the 2017-2021 period, research moved from primary to applied, with emerging concepts such as cognitive behavioral therapy. Since 2022, cognitive behavioral therapy has remained the main research topic, whereas the concept of mHealth has not received much attention.

#### Analysis of the Software Development Stages Described in the Literature

In terms of the software development stages described in the literature, of the 75 papers, 49 (65%) referred to the design stage, 19 (25%) pertained to the clinical validation stage, and 7 (9%) concerned the marketing stage. This distribution reflects the negligible development of software for tinnitus treatment. Preliminary research found that the actual demand for such software was notably high.

#### Analysis of the Core Treatment Techniques Described in the Literature

Upon further analysis, it was found that 50 (67%) of the 75 papers primarily described treatments that used specific software. Of the remaining 25 studies, 12 (48%) mentioned only internet-based or web-based assessment tools for tinnitus, without any specific treatment associated. Of these 12 publications, 6 (50%) described mobile transient tinnitus assessment, and 4 (33%) used machine learning for predicting tinnitus, whereas 1 (8%) described a repository of tinnitus information, and 1 (8%) described a method for characterizing tinnitus heterogeneity. Of the 50 articles describing treatments, the mentioned treatments were mainly in the categories of cognitive behavioral therapy and sound therapy, with 30 (60%) and 13 (26%) papers, respectively. Of the remaining 7 studies, 1 (14%) included usual service therapy using artificial intelligence (AI) to expose the patient to the tinnitus environment, 1 (14%) used a tinnitus e-plan (an intervention coding method), 1 (14%) used visualized mobile electroencephalogram (EEG) for tinnitus detection, 1 (14%) used meditation (to help patients relieve tinnitus), 1 (14%) used both cognitive behavioral therapy and sound therapy, and 2 (29%) used a game training method related to the localization and perception of a sound source as well as auditory attention.

We compared the stage of software development reported in the literature with the therapies related to the respective software. We found that only a single software using cognitive behavioral therapy and sound therapy was in the market stage. With regard to software in the clinical validation stage, 9 (32%) of the 28 apps used cognitive behavioral therapy tools, 3 (12%) used sound therapy, and 2 (17%) used game training methods related to sound source localization perception and auditory attention. Of the 29 apps described in the literature that were in the design phase, 18 (62%) used cognitive behavioral therapy, 7 (24%) used sound therapy, 1 (3%) used habituation therapy (in which AI exposes the patient to the tinnitus environment), 1 (3%) used meditation, 1 (3%) used visualized mobile EEG, and 1 (3%) used an intervention coding approach.

### Analysis of the Listed Apps

#### Analysis of the Number of Apps

Mobile tinnitus treatment software is currently in high demand. In this study, 28 apps were identified from software data sources (Figure 2), 4 of which were from software with specific names in the literature. These 28 apps consisted of 18 (64%) iOS apps and 10 (36%) Android apps (Multimedia Appendix 4). Most of these apps were developed by institutions (22/28, 79%); a few were developed by individuals (2/28, 7%). Among the 24 apps obtained from product data, 13 (54%) were medical apps, and 9 (38%) were health apps.

Regarding the software providers, of the 28 apps, 22 (79%) were available on the Apple App Store (China), and 21 (75%) were available on the Apple App Store (United States). In addition, 5 (18%) were available on the Google Play Store, and 5 (18%) were available on the Huawei, Xiaomi, Oppo, and Vivo marketplaces. Software activity can be reflected at the time of listing and at the time of the latest update. After data collection, we found that in 23 (82%) of the 28 apps, time information was complete.

#### Analysis of the Distribution of Source Countries

Multimedia Appendix 5 depicts the origin of the software developers. Of the 28 apps, 10 (36%) were developed in the United States, 8 (29%) in China, and 9 (32%) in other countries (n=1, 11% in the United Kingdom; n=3, 33% in Germany; n=3, 33% in Denmark; n=1, 11% in Poland; and n=1, 11% in New Zealand). Of the 28 apps, there was 1 (4%) whose country of origin could not be found. Regarding specific locations, the US software developers hailed from 7 states (3/10, 30% from Minnesota; 2/10, 20% from Texas; 1/10, 10% from Colorado; 1/10, 10% from Wisconsin; 1/10, 10% from California; 1/10, 10% from Florida; and 1/10, 10% from Oregon). Of the 8 software developers based in China, 7 (88%) were from regions within mainland China (n=3, 43% from Beijing; n=2, 29% from Jiangsu; n=1, 14% from Shanghai; and n=1, 14% from Guangdong), and 1 (13%) was from Hong Kong, China.

#### Analysis of the Functional Division of the Apps

When analyzing the 28 tinnitus treatment–related apps, this study divided them into three main categories: (1) screening and evaluation, (2) intervention and rehabilitation, and (3) education and information. Of the 28 software programs, 28 (100%) were related to intervention and rehabilitation, 11 (39%) were related to screening and evaluation, and 5 (18%) were related to education and information. Regarding the users of the software, of the 28 apps, 26 (93%) were intended to be used by patients or their families, and 2 (7%) were intended to be used by medical staff. In this study, we considered whether a professional was required to assist patients with the software and found that, in the case of 7 (25%) of the 28 apps, a professional was needed to assist the patient. By contrast, the remaining apps (21/28, 75%) could be used by patients without assistance from a professional.

When analyzing the specific functions of the apps, this study divided them into the following 6 areas: assessment, advice, detection, counseling, treatment, and relief. Of the 28 apps, 6 (21%) were dedicated to patient assessment, 4 (16%) could provide advice based on the actual patient situation, 5 (18%) could perform a preliminary test of the patient’s tinnitus or hearing condition, another 5 (18%) had a web-based counseling function, and 13 (46%) could provide the user with a treatment plan for the different tinnitus conditions. The primary product information is described in Multimedia Appendix 6.

#### Analysis of Treatments Provided by the Apps

After examining the treatments mentioned in the app descriptions, we found that, of the 28 apps, 7 (25%) used masking therapy, 2 (7%) used habituation therapy, and 2 (7%) used cognitive behavioral therapy. Moreover, of the 28 apps, 1 (4%) used sound therapy, 1 (4%) used neuromusic therapy, 1 (4%) used play therapy and frequency discrimination therapy, 1 (4%) used transcallosal vagus nerve microcurrent stimulation, and 1 (4%) used neuromodulation therapy. The remaining apps (12/28, 43%) did not specify the type of treatment used.

#### Analysis of App Validity

Regarding the validity and reliability of the apps, we found that some of the apps (6/28, 21%) were created by developers in collaboration with specific authorities. The 6 partner organizations are the American Tinnitus Association; the Eye, Ear, Nose, and Throat Hospital affiliated to Fudan University (Shanghai, China); a team of doctors from Tsinghua University (Beijing, China); a team of tinnitus specialists from well-known tertiary hospitals and first-line tinnitus experts; a Canadian team with Food and Drug Administration (FDA)–approved tinnitus rehabilitation core technology; and the University of California (Los Angeles, California, United States).

### Comparative Analysis of Literature and Apps

Regarding the target audience, the software described in the literature was mostly consistent with the marketed apps. Most of the apps (26/28, 93%) focused on patients and their families, whereas a few (2/28, 7%) targeted medical staff. Most of the apps (20/28, 71%) provided different types of sounds to relieve tinnitus. A few of the apps (5/28, 18%) required the use of hearing aids, being aimed at medical staff use.

In the comparative analysis of the app scenarios, there was a strong consistency between the 2 types of apps. Most of the apps focused on the home scenario (22/28, 79%), whereas a few of the apps (6/28, 21%) are meant to be used in hospitals or other institutions dedicated to professional audiology. The tinnitus treatment apps currently available for download and use are mostly rudimentary. Most of them (22/28, 79%) can only provide initial relief to the patients or provide an assessment of their condition

In a comparative analysis of the treatment techniques, the software described in the literature was predominantly based on sound therapy. This was a preferred approach, probably because, on the one hand, it was easier to implement in combination with mHealth software and, on the other hand, it was effective in tinnitus treatment. Half of the apps (16/28, 57%) listed in the app stores were based on acoustic and cognitive behavioral therapy, with significant user feedback available. Taking advantage of the psychoemotional characteristics of patients with tinnitus, a potential treatment method for tinnitus that involves transcranial magnetic electrical stimulation (containing 2 models: one for transcaudal vagus nerve microcurrent stimulation technology and one for neuromodulation therapy) needs to be developed. The other half (12/28, 43%) of the available apps did not describe the treatment method and lacked user evaluations. This was probably because of the relatively early stage of development of such apps and their limited user results.

A comparative analysis from a clinical standpoint showed that the apps mentioned in the literature were in the theoretical design stage. Relevant studies involving the application to human patients generally need ethics review committee approval, have to meet high product quality requirements, and have to sustain through long development and validation pathways, as well as overcome low translation rates. Individual apps are not marketed because they are not currently registered, despite having been rigorously designed at the theoretical level. Among the marketed apps, the proportion of those in the therapeutic intervention stage is high (5/7, 71%), with a relatively strong practicality and a modest performance.

## Discussion

### Current Status and Trends in Research

#### Literature Information

The number of studies in the field of tinnitus apps has increased yearly, with a rapid growth after 2015 and with the annual publication volume peaking in 2021. The articles were published in journals of moderate quality. Nevertheless, the acceptance rates of articles in the field by top medical journals were low. Of the 75 research articles in this field, 51 (68%) were included in the Web of Science core collection and had an average journal impact factor of 5.12 (spanning between 1.245 and 25.617 in the field of psychotherapy and psychosomatics). The studies were mainly published in the *American Journal of Audiology* and in the *Journal of the American Academy of Audiology*, with 18 (n=12, 67% and n=6, 33%, respectively) articles published as of February 2022, representing 24% (18/75) of the total number of articles. Nevertheless, the acceptance rates of articles in this field in top medical journals were poor, with no relevant studies published in the *New England Journal of Medicine*, *The Lancet*, the *Journal of the American Medical Association*, or *The BMJ*. The annual output of the most productive authors was the highest in 2021. In accordance with the Lotka law, this study identified 16 highly productive authors in the field, all of whom have published consistently in the last 3 years. This suggests that this area is steadily and rapidly growing. The authors were mainly from Germany (19/75, 25%), the United Kingdom (16/75, 21%), and the United States (12/75, 16%), with fewer authors from other countries (27/75, 36%), and only a single author from China (1/75, 1%). Of the 75 articles, 47 (63%) were published by authors from Germany, the United Kingdom, and the United States.

The current hot spot in this field is the research on sound and cognitive behavioral therapy, focusing on the outcomes for the treatment of patients with tinnitus. Thematic map analysis showed that the terms in the second quadrant of the thematic map included cognitive behavioral therapy, sound therapy, and tinnitus treatment outcomes. This indicates that research on cognitive behavioral therapy and sound therapy (with a focus on tinnitus treatment outcomes) is a current hot spot in the field of mHealth-based software interventions for tinnitus treatment. This area of research is well developed, with a focus on the assessment of tinnitus, but its importance is not sufficiently recognized yet. Our results are in agreement with those of relevant studies conducted in recent years [22].

#### App Information

Most of the apps (16/28, 57%) in this field have been newly developed and provide functional support for therapeutic interventions for tinnitus. Nevertheless, software development in this field lacks a research basis. Such software often has high requirements for ancillary apps [23], such as the sound quality and calibration of headphones. Cognitive behavioral therapy relies on professional tinnitus specialists for guidance. By contrast, it is difficult to achieve an accurate grasp of a user’s criteria for the use of such apps with mobile phone sensors or wearable apps alone [24]. As a result, and in comparison with other fields, the number of diagnostic apps is relatively small.

The adoption of apps under development has been poor, with the vast majority of the available apps used in very few or no real-life scenarios. The results showed that only 28 designed apps were available for download from app stores. This limitation suggests that, although many tinnitus treatment–related apps have been developed by experts and academics in recent years, most of them still lack practical use. It also suggests that these apps have a very homogeneous collection of patient information and a single-center validation of their effectiveness; in addition, they lack validity studies for clinical retesting as well as user feedback [25]. The reasons behind these aspects could be related to other attributes of the apps, such as their effectiveness and ease of use. To develop and validate apps that can be used in the clinical setting, researchers should also focus on other attributes, such as the ease of use of the apps.

#### Translation Status

For many years, there has been a lack of translation of the results of tinnitus treatment interventions using mHealth software and apps. This lack of translation is consistent with the results of our study on the analysis of product applications in this area. The vast majority of these apps (22/26, 85%) require both research and development, with scientific validity and effectiveness in a state of urgent need, and are far from truly achieving their goal of an effective tinnitus treatment intervention [26].

### Problems and Potential

#### The Overall Picture of Tinnitus Treatment Apps Is Unsatisfactory

##### Scoring Analysis of Tinnitus Treatment Apps

Regarding the ratings of the included 28 tinnitus treatment–related apps (n=24, 86% from product data and n=4, 14% from literature data) on the Qimai app data analysis platform, 17 (61%) had ratings as high as 5.00 out of 5.00 and as low as 1.00 out of 5.00 (with an average rating of 3.84 out of 5.00). Of these 17 rated apps, China and the United States accounted for 7 (41%) and 5 (29%), respectively. The 5 apps developed in the United States had ratings of 4.40, 4.80, 4.80, 4.90, and 5.00 (average rating: 4.78), whereas the 7 apps developed in China had ratings of 1.00, 1.80, 3.80, 4.00, 4.10, 4.30, and 4.70 (average rating: 3.39).

##### Evaluation Analysis of Tinnitus Treatment Apps

With regard to the information on the 28 tinnitus treatment–related apps provided on the Qimai app data analysis platform, 10 good reviews were collected over 1 year. All these reviews referred to 6 (21%) of the 28 apps and were about their usefulness and usability. After analyzing the geographical regions of origin, our study found that the developers of 5 (83%) of these 6 apps were from China, with 5 positive reviews and 3 negative reviews.

##### Traditional Treatment Methods Are Far From Satisfactory

There is currently no international uniform treatment protocol for tinnitus. Moreover, evidence-based medical research has not found a single method with definitive efficacy for all types of tinnitus. The available methods include medications, repetitive transcranial magnetic stimulation, electrical stimulation of the tympanic capsule, cochlear implants, electrical stimulation of the vagus nerve, and acupuncture [27]. The treatment of tinnitus relies on obtaining an accurate hearing profile of the patient as well as indicators of the central frequency and intensity of the sound produced by tinnitus. After excluding the etiologies associated with tinnitus, counseling, cognitive behavioral therapy, and sound therapy have become the main treatment approaches [28]. Other approaches include relaxation and sequential therapy [29]. The lack of adjunctive therapies to improve outcomes in complex conditions is a common problem in tinnitus treatment, recognized both domestically and internationally.

##### Clear Differences in Tinnitus Treatment Apps at the Research Level Versus the App Level

Current research has uncovered the lack of connection between mHealth apps and their applications. In recent years, national and international research scholars have recognized the roles of neural synchronization and remodeling the mechanisms of action in tinnitus [30]. On the basis of these findings, adjunctive sound therapy involves the administration of appropriate acoustic stimulation (of a specific frequency and duration) to break abnormal nerve synchronization and remodeling. This procedure seeks to form a new auditory center, thereby reducing or eliminating tinnitus [31]. The use of mHealth apps to provide appropriate auditory training for patients with tinnitus has proven effective at the research level. Nevertheless, important aspects are lacking and need to be addressed. These include a scientific basis for software function, the standardization and calibration of the sounds emitted, real-time guidance from experts during the use of the apps by patients, clinical validation and feedback from patients on product effectiveness, and the connection between research and apps in this field.

##### Significant Differences in the Status of Relevant Research and Apps Between China and Other Countries

Our study showed that, although most research and apps in this area were developed outside China, there are still important limitations. The sample size of research and apps in this field, both domestically and abroad, was small. The final number of apps found to meet all inclusion criteria was only 28, of which only 4 (14%) were documented in the literature. This limitation has a particular impact on the results of our study. Moreover, it also reflects the decreased volume of research in this field, which strengthens the innovation and value of our work.

#### Tinnitus Is Difficult to Treat and Requires Urgent Assistance From Emerging Technologies

With the increased stress levels in modern work and life, tinnitus does not appear in isolation. Nevertheless, it is a combination of symptoms closely related to the etiological mechanisms and aggravating factors of several systemic diseases. More than 50% of patients with tinnitus experience sleep disturbances [32]. In the United Kingdom, the prevalence rate of tinnitus in the adult population is 10.1%, with the disorder severely affecting the quality of daily life [33]. In the United States, the prevalence rate of tinnitus in adolescents is from 7% to 9%, and in older people, it is approximately 25% [34]. In Japan, the prevalence rate of tinnitus in the adult population is 11.9% [35]. Although there are no large-scale epidemiological data on the prevalence rates of tinnitus in China, available studies have reported its prevalence rate in the adult population to be approximately 10%. Approximately 5% of the patients with tinnitus seek medical treatment, and approximately 2% of the patients have tinnitus that severely affects their life, sleep, the ability to concentrate and work, and social activities [36]. The complex and heterogeneous etiology of tinnitus as well as its unclear pathogenesis have contributed to the poor treatment outcomes for this disorder. Nevertheless, as industry and technology continue to develop and society ages, the prevalence of tinnitus is gradually increasing, making its early detection, diagnosis, and effective treatment urgent.

The evidence-based guidelines for clinicians managing patients with tinnitus [1] recommend an acoustic therapy approach, in which any sound can be used to alter the perception of, and response to, tinnitus, for the patient’s benefit. This therapy can be performed with environmental optimization devices such as hearing aids, sound generators, and tinnitus hybrid acoustic therapy devices, as well as commonly used devices such as mp3 players, smartphones, and radios. Tinnitus rehabilitation approaches must not disregard the integration of traditional methods with innovative technologies [37]. With the expansion and increased application of the mobile internet in various industries, health care–focused mobile internet technology has been receiving increased interest owing to its rapid development. Therefore, the combination of sound therapy and cognitive behavioral therapy for tinnitus has led to the development of an mHealth app model with this purpose.

### Future Developments and Outlook

#### The Theory of mHealth for Adjunctive Treatment and Intervention in Tinnitus Is Logically Clear and Scientifically Solid

To address the ineffectiveness of traditional treatments for tinnitus, one of the 3 most difficult conditions plaguing human health in the otolaryngology field (the other 2 being deafness and vertigo), researchers have been exploring interdisciplinary avenues [38]. The evidence-based guidelines for clinicians managing patients with tinnitus recommend cognitive behavioral therapy for patients with chronic compensatory tinnitus, as well as effective and feasible acoustic treatment options provided by physicians or hearing aids for patients with tinnitus with hearing loss [39]. mHealth is unique in providing behavioral health interventions. One of its scientific theories is cognitive behavioral therapy. The future integration of mHealth for tinnitus treatment and intervention is in line with the recommendations of professional guidelines and has a sound scientific and theoretical basis.

#### The Pathway for mHealth to Be Used as an Adjunctive Treatment and Intervention in Tinnitus Is Clear and Highly Feasible

One of the theoretical foundations of mHealth is cognitive behavioral therapy. As of 2016, approximately 500 mHealth apps based on cognitive behavioral theory were available in the Apple App Store and Google Play Store. Current mobile apps are relatively well established in the use of cognitive behavioral therapy interventions in psychosocial health services to relieve anxiety, depression, and stress as well as treat other physical ailments and improve maladaptive behavioral problems (such as eating disorders and substance abuse). As a result, the product experience in mHealth is relatively mature. More than half of all patients with tinnitus have comorbid psychological problems such as anxiety, depression, and sleep disorders [40]. On the basis of the existing experience with mHealth, there is a clear and feasible pathway for adjunctive treatment and intervention in tinnitus.

#### The Use of mHealth for Adjunctive Treatment and Intervention in Tinnitus Is Effective and Has Good Generalizability

Our findings regarding mHealth use in mental health, disease management, and health behavior promotion indicate that mHealth apps, combined with cognitive behavioral therapy, were highly effective in reducing tinnitus pain, anxiety, and depression as well as in improving the patients’ quality of life. In 2019, Aazh et al [41] found that the use of cognitive behavioral therapy for tinnitus was more effective when used 8 to 24 times per week for 60 to 120 minutes each time. The treatment could be remotely guided through an mHealth App to accommodate more patients and promote research progress on effective tools for treating tinnitus [42].

### Limitations

Our study has some limitations. First, the data used were mainly from published literature (which included only scientific literature). Second, this study performed a simplified analysis of the product translation. We only analyzed the overall conversion rate and did not analyze specific apps and specific application scenarios. Finally, owing to the difficulty of obtaining complete data on apps and scientific research funds, we have only preliminarily explored the input-output relationship of some of the apps.

### Conclusions

The field of monitoring, diagnosing, and treating tinnitus disorders using mHealth apps has shown rapid overall growth during recent years. Accordingly, there has been an increase in the number of relevant publications per year. Progress has been made in research on cognitive behavioral therapy, with a focus on the improvement of tinnitus symptoms and on the development of apps to support tinnitus monitoring and interventions. Nevertheless, the number of studies in this area is still low, international collaborations are lacking, the acceptance rates of articles in this field in refereed medical journals are poor, and most of the developed apps are not used in real-world settings. Future research should address the need for increasing tinnitus awareness and strengthening international collaborations to achieve an improved monitoring of tinnitus, using novel AI tools. In addition to the validation of tinnitus treatment apps, future research should also focus on the app properties that can promote the application of such apps in the real world.

## Appendix

Multimedia Appendix 1 Query strategy and results.

Multimedia Appendix 2 Inclusion and exclusion criteria applied to select the target literature.

Multimedia Appendix 3 The evolution in the annual number of relevant studies published over the years.

Multimedia Appendix 4 Description of basic app information.

Multimedia Appendix 5 Distribution of software sources.

Multimedia Appendix 6 Distribution of source countries.

## Abbreviations

AI: artificial intelligence
EEG: electroencephalogram
FDA: Food and Drug Administration
mHealth: mobile health
PRISMA: Preferred Reporting Items for Systematic Reviews and Meta-Analyses

## Data Availability

This study was conducted using public databases. Users can download relevant data free of charge.
