# Supplementary material for: Current Status and Trends in mHealth-Based Research for Treatment and Intervention in Tinnitus: Bibliometric and Comparative Product Analysis
Source: JMIR Mhealth Uhealth. 2023 Aug 24;11:e47553. doi: 10.2196/47553 (PMC10485709; doi:10.2196/47553)
Supplement: Multimedia Appendix 1 [file mhealth_v11i1e47553_app1.docx]

**Multimedia Appendix 1: Query strategy and results**

| Databases | Query | Results | Query time |
| --- | --- | --- | --- |
| PubMed | ('tinnitus'/exp OR tinnitus) AND ('software'/exp OR software OR 'application'/exp OR application OR app OR Internet* OR platform OR 'programmer'/exp OR programmer) | 1373 | 2022.2.28 |
| Embase | ('tinnitus'/exp OR tinnitus) AND ('software'/exp OR software OR 'application'/exp OR application OR app OR Internet* OR platform OR 'programmer'/exp OR programmer) | 1301 | 2022.2.28 |
| ACM | [All: tinnitus] AND [[All: software] OR [All: application] OR [All: app] OR [All: Internet*] OR [All: platform] OR [All: programmer]] | 86 | 2022.2.28 |
| IEEE | (tinnitus) AND (Software OR application OR app OR Internet* OR platform OR programmer) | 34 | 2022.2.28 |
| WoS core  collection | TS=tinnitus AND TS=(Software OR application OR app OR Internet* OR platform OR programmer) | 1591 | 2022.2.28 |
