# Supplementary material for: Current Status and Trends in mHealth-Based Research for Treatment and Intervention in Tinnitus: Bibliometric and Comparative Product Analysis
Source: JMIR Mhealth Uhealth. 2023 Aug 24;11:e47553. doi: 10.2196/47553 (PMC10485709; doi:10.2196/47553)
Supplement: Multimedia Appendix 2 [file mhealth_v11i1e47553_app2.docx]

**Multimedia Appendix 2: Inclusion and exclusion criteria of target literature.**

| Criterion name | Declaration |
| --- | --- |
| Inclusion  criterion 1’ (IC1’) | Article describing tinnitus or tinnitus-related disorders. |
| Inclusion  criterion 2’ (IC2’) | Articles related to the evaluation, testing, development, use or application of software applications, platforms. |
| Exclusion criterion 1’ (EC1’) | Overviews, reviews, letters, publications, commentaries. |
| Exclusion criterion 2’ (EC2’) | Non-English articles. |
| Exclusion criterion 3’ (EC3’) | Articles where access to the full text is not allowed. |
| Exclusion criterion 4’ (EC4’) | The article only describes the disease, no software was designed. |
| Exclusion criterion 4’ (EC5’) | The software studied does not directly address tinnitus. |
