# Supplementary figures and images for: Current Status and Trends in mHealth-Based Research for Treatment and Intervention in Tinnitus: Bibliometric and Comparative Product Analysis
Source: JMIR Mhealth Uhealth. 2023 Aug 24;11:e47553. doi: 10.2196/47553 (PMC10485709; doi:10.2196/47553)

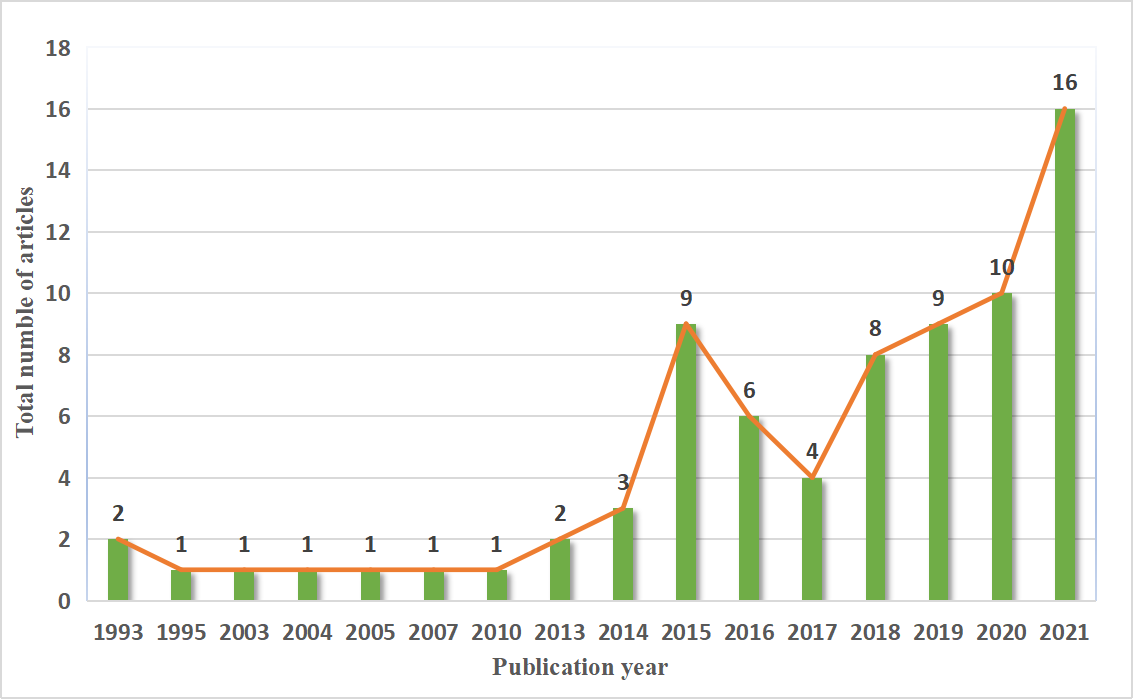

Supplement: Multimedia Appendix 3 [file mhealth_v11i1e47553_app3.png]

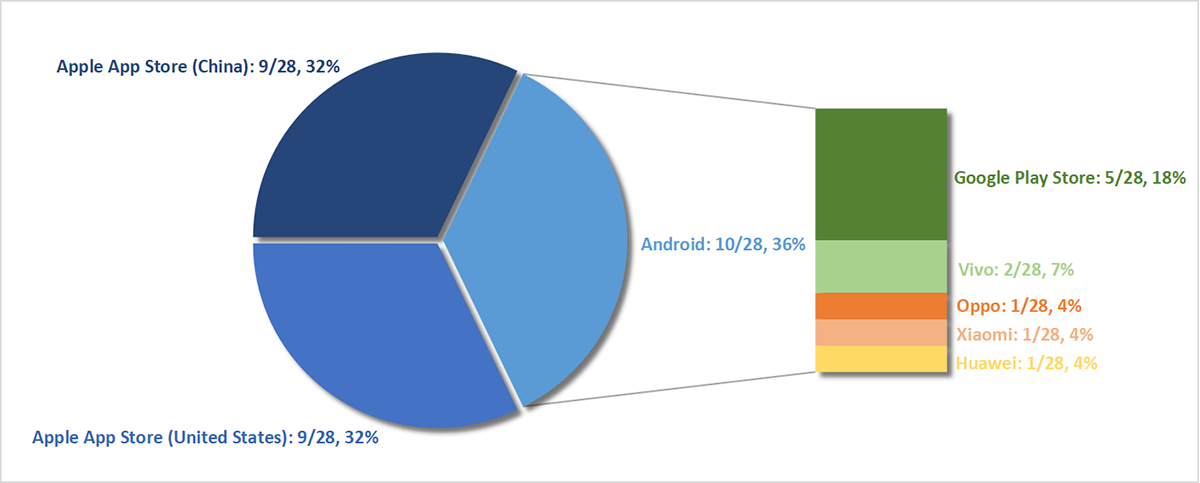

Supplement: Multimedia Appendix 4 [file mhealth_v11i1e47553_app4.png]

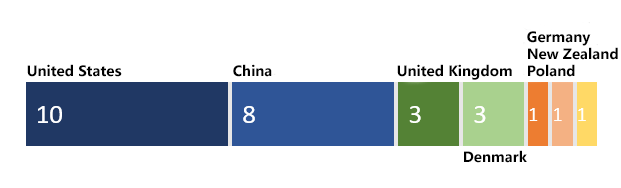

Supplement: Multimedia Appendix 5 [file mhealth_v11i1e47553_app5.png]

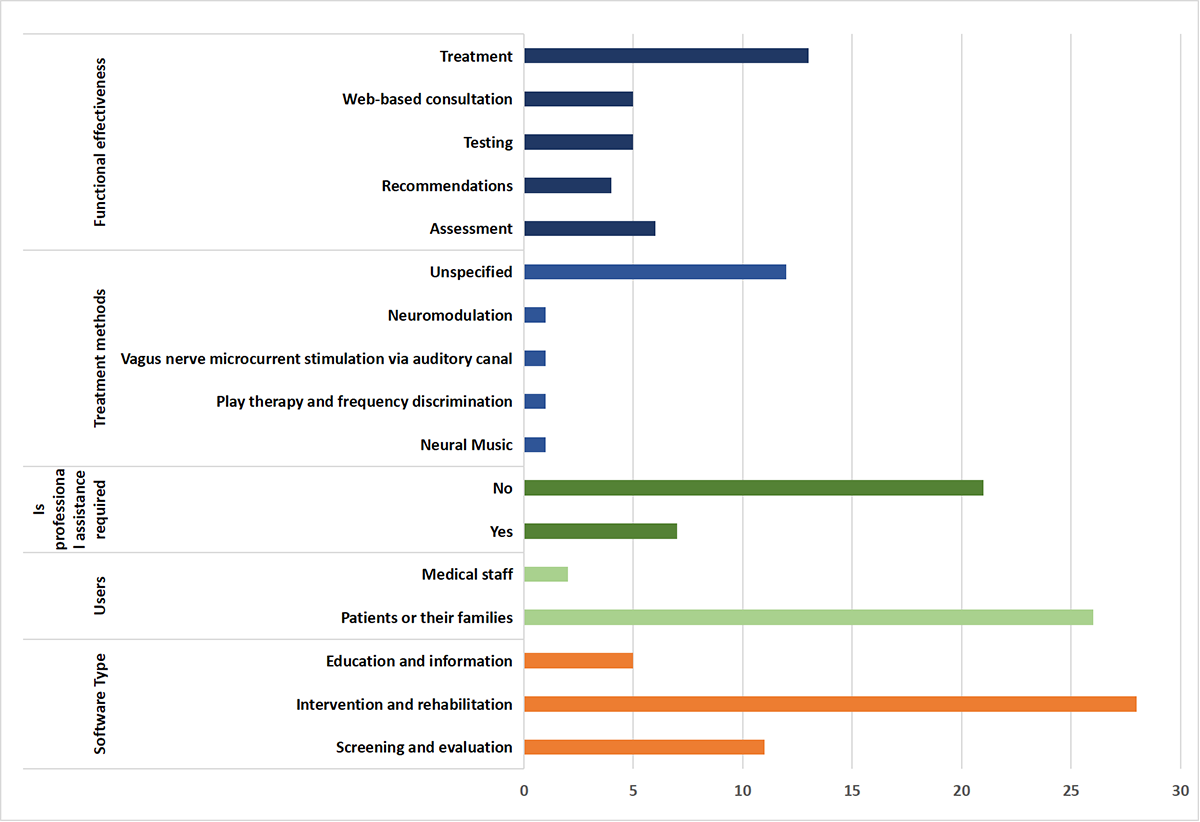

Supplement: Multimedia Appendix 6 [file mhealth_v11i1e47553_app6.png]
